# Days Alive and Out of Hospital as an Outcome Measure in Patients Receiving Hyperacute Stroke Intervention

**DOI:** 10.1161/JAHA.123.032321

**Published:** 2024-07-03

**Authors:** Joseph Donnelly, Jae Beom Hong, Luke Boyle, Vivien TY Yong, William K. Diprose, Juliette Meyer, Douglas Campbell, P. Alan Barber

**Affiliations:** ^1^ Department of Medicine University of Auckland Auckland New Zealand; ^2^ Department of Neurology Auckland City Hospital Auckland New Zealand; ^3^ Department of Statistics University of Auckland Auckland New Zealand; ^4^ Department of Anaesthesiology University of Auckland Auckland New Zealand

**Keywords:** days alive and out of hospital, endovascular thrombectomy, outcomes, Rankin score, stroke, Ischemic Stroke, Cerebrovascular Disease/Stroke, Mortality/Survival, Quality and Outcomes

## Abstract

**Background:**

Patient outcome after stroke is frequently assessed with clinical scales such as the modified Rankin Scale score (mRS). Days alive and out of hospital at 90 days (DAOH‐90), which measures survival, time spent in hospital or rehabilitation settings, readmission and institutionalization, is an objective outcome measure that can be obtained from large administrative data sets without the need for patient contact. We aimed to assess the comparability of DAOH with mRS and its relationship with other prognostic variables after acute stroke reperfusion therapy.

**Methods and Results:**

Consecutive patients with ischemic stroke treated with intravenous thrombolysis or endovascular thrombectomy were analyzed. DAOH‐90 was calculated from a national minimum data set, a mandatory nationwide administrative database. mRS score at day 90 (mRS‐90) was assessed with in‐person or telephone interviews. The study included 1278 patients with ischemic stroke (714 male, median age 70 [59–79], median National Institutes of Health Stroke Scale score 14 [9–20]). Median DAOH‐90 was 71 [29–84] and median mRS‐90 score was 3 [2–5]. DAOH‐90 was correlated with admission National Institutes of Health Stroke Scale score (Spearman rho −0.44, *P*<0.001) and Alberta Stroke Program Early CT [Computed Tomography] Score (Spearman rho 0.24, *P*<0.001). There was a strong association between mRS‐90 and DAOH‐90 (Spearman rho correlation −0.79, *P*<0.001). Area under receiver operating curve for predicting mRS score >0 was 0.86 (95% CI, 0.84–0.88), mRS score >1 was 0.88 (95% CI, 0.86–0.90) and mRS score >2 was 0.90 (95% CI, 0.89–0.92).

**Conclusions:**

In patients with stroke treated with reperfusion therapies, DAOH‐90 shows reasonable comparability to the more established outcome measure of mRS‐90. DAOH‐90 can be readily obtained from administrative databases and therefore has the potential to be used in large‐scale clinical trials and comparative effectiveness studies.

**Nonstandard Abbreviations and Acronyms**
ASPECTS: Alberta Stroke Program Early CT Score
DAOH: days alive and out of hospital
DAOH‐90: days alive and out of hospital at 90 days
EVT: endovascular thrombectomy
IVT: intravenous thrombolysis
mRS: modified Rankin Scale
mRS‐90: modified Rankin Scale at 90 days
NIHSS: National Institutes of Health Stroke Scale

**Clinical Perspective**

**What Is New?**

- Days alive and out of hospital at 90 days is an objective outcome measure that counts the number of days in the 90 days since an index event that a patient is both alive and out of hospital, and, in contrast to the modified Rankin Scale score, can be obtained from large administrative data sets.
- In this study of patients with stroke treated with reperfusion therapies, days alive and out of hospital at 90 days showed comparability to the more established outcome measure of the modified Rankin Scale.

**What Are the Clinical Implications?**

- Days alive and out of hospital has the potential to be used in large‐scale clinical trials and comparative effectiveness studies without the need for direct patient contact.

Measuring patient outcome after stroke is essential for audit, quality improvement, and clinical research. The modified Rankin Scale (mRS) score is one of the standard patient outcome measures in stroke.^1^, ^2^ The mRS has advantages such as its established evidence for valid use, it is a graded measure of outcomes, and it can be conducted either in face‐to‐face settings or via a structured telephone interview. However, the mRS has significant interrater variability,^3^ and in large‐scale trials is less practical due to the requirement of dedicated individual patient follow‐up.

Days alive and out of hospital (DAOH) is a measure of patient outcome used in cardiovascular and surgical populations that circumvents some of the limitations of the mRS.^4^, ^5^, ^6^, ^7^ DAOH counts the days alive and out of hospital since a reference time point such as hospital admission or index surgery, until a defined follow‐up period with 30 days, 90 days, or 1 year being common reference time points. Time in hospital includes time in rehabilitation facilities or residential care, with noncontiguous time in these settings summed, for example, hospital readmission within the set follow‐up period. Similar end points called “home days,” “home time,” or “days alive at home” have been compared with mRS in population‐based studies in stroke, used as a secondary end point in a multicenter stroke trial, and as a primary end point in a trial of haloperidol for the treatment of delirium.^8^, ^9^, ^10^ DAOH has several advantages over mRS in that it is less labor intensive to collect, economical, and in many cases can use component data that area already routinely collected. However, its use as a primary outcome measure in the hyperacute stroke setting so far has been limited.

We aimed to provide evidence for valid use of DAOH at 90 days (DAOH‐90) after acute stroke reperfusion therapy by comparing it with known prognostic and treatment factors, complications and mRS score at 90 days (mRS‐90).

## Methods

Summary data that support the findings of this study are available from the corresponding author upon reasonable request. Anonymized individual patient data from the New Zealand National Minimum Data Set can be sought from the Ministry of Health with appropriate regulatory approval.^11^ This study was approved by the regional ethics committee (21/CEN/157) and waiver of informed consent was granted.

Consecutive patients with ischemic stroke treated with intravenous thrombolysis (IVT) or endovascular thrombectomy (EVT) were included. Patients were excluded if they had missing clinical, radiographic, or patient outcome data. The study was carried out at a comprehensive stroke center that provides EVT services to a population of approximately 2.8 million people. All patients treated with IVT and EVT are entered into a mandatory national registry, which contains data regarding patient demographics, medical comorbidities, clinical features, treatment details, and patient outcomes. The attending clinical teams make treatment decisions such as the eligibility for IVT or EVT. Stroke onset was defined as the time the patient was last known well. IVT was with alteplase at 0.9 mg/kg with a thrombolysis window of 4.5 hours until the publication of the EXTEND (Extending the Time for Thrombolysis in Emergency Neurological Deficits) study when an expanded thrombolysis time window was used.^12^ An EVT treatment window of 6 hours was used until publication of the DAWN (DWI [Diffusion‐Weighted Imaging] or CTP [Computed Tomography Perfusion] Assessment With Clinical Mismatch in the Triage of Wake‐Up and Late Presenting Strokes Undergoing Neurointervention With Trevo) and DEFUSE (Endovascular Therapy Following Imaging Evaluation for Ischemic Stroke) studies.^13^, ^14^

Baseline and 24‐hour National Institute of Health Stroke Scale (NIHSS) scores were assessed by the treating clinical team. The degree of ischemic change was determined using the Alberta Stroke Program Early CT Score (ASPECTS) by 2 of the investigators (W.D., J.B.H.). ASPECTS is a quantitative topographic score for middle cerebral artery vascular territory strokes that assigns 10 points to a normal scan and deducts 1 point for any of the 10 middle cerebral artery subregions that show ischemic change.^15^

DAOH is an outcome measure that combines the 2 constructs of mortality and days out of hospital and was calculated as previously described.^16^ Data on all public and private health facility admissions were obtained from the National Minimum Data Set, a New Zealand Ministry of Health supported database with demographic, process, and outcome data for all hospital episodes. Data transfer is completed within 3 weeks of hospital discharge with >99% of hospital episodes captured.^11^ The amount of time spent alive and out of hospital was calculated from the day of hospital admission to 90 days using a custom R script.

The mRS combines a number of constructs within its 7 categories including disability, self‐care, ambulation, dependence or burden of care, and mortality.^17^ mRS‐90 scores were obtained from a structured telephone or in‐person interview by a trained assessor. Functional independence was defined as mRS score 0, 1, or 2 at day 90. Early neurological improvement was defined as a decrease in NIHSS score of ≥8 between hospital admission and 24 hours, or an improvement to 0 or 1 at 24 hours. Cause of stroke was determined according to the Trial of Org 10 172 in Acute Stroke Treatment score.^18^ Successful recanalization was defined as a modified Thrombolysis in Cerebral Infarction score of 2b to 3. Safety outcomes included symptomatic intracranial hemorrhage on 24‐hour CT (defined by the safe implementation of thrombolysis in stroke monitoring study definition) and mortality at 90 days.^19^

The document, *Standards for Educational and Psychological Testing*, provides a framework for assessing the valid use of DAOH‐90 in this context.^20^ Specifically, the relationship between DAOH‐90 and demographic, clinical, and treatment prognostic variables was considered as “comparative validity evidence,” and between DAOH‐90 and mRS as “evidence regarding relationships with criteria.”

### Statistical Analysis

The relationship between baseline prognostic factors and DAOH were performed using a Spearman correlation coefficient for continuous or ordinal data or a Mann–Whitney test for binary variables. The relationship between mRS‐90 and DAOH‐90 was assessed using a Spearman correlation on the whole cohort and within relevant clinical subgroups. Receiver operating characteristic analysis was performed to assess the discriminative ability of DAOH‐90. Multivariable models were built on a subcohort (n=871) using previously established predictors of outcome that were available in the current cohort, noting ASPECTS score was calculated only in anterior circulation EVT cases.^21^ Patients with missing data for specific variables were excluded when that particular variable was being tested (pairwise deletion). All tests were 2 tailed, and *P*<0.05 was considered significant. All statistical analysis was performed on R (R studio v4.1.2, Vienna).^22^

## Results

The study included 1278 patients with stroke (714 male, median age 70 years [interquartile range 59–79], baseline NIHSS score 14 [9, 20]) treated with acute stroke reperfusion therapies (530 EVT only, 264 IVT only, 484 both) between January 1, 2015 and December 31, 2021 (Figure S1). Demographic and clinical features of the cohort are described in Table 1. The median DAOH‐90 was 71 [29, 84] days, and was not normally distributed with peaks at 0 and 85 days (Figure S2). The distributions differed across groupings of age, admission NIHSS score, and ASPECTS (Figure S3). 691 (54%) patients were functionally independent at day 90 day.

**Table 1:** Demographic and Clinical Description of Cohort

No.	1278
Age, y (median [IQR])	70 [59, 79]
Sex, male (%)	714 (56)
Diabetes (%)	249 (19)
NA	8 (1)
Ischemic heart disease (%)	234 (18)
NA	7 (1)
Atrial fibrillation (%)	586 (46)
NA	6 (0)
Hypertension (%)	807 (63)
NA	7 (1)
Trial of Org 10 172 in Acute Stroke Treatment (%)	
Cardioembolic	614 (48)
Large artery atherosclerosis	250 (20)
Other determined cause	55 (4)
Small vessel occlusion	55 (4)
Undetermined cause	292 (23)
NA	12 (1)
Admission National Institute of Health Stroke Scale score (median [IQR])	14 [9, 20]
Alberta Stroke Program Early CT [Computed Tomography] Score (median [IQR])	8 [7, 10]
Intervention (%)	
EVT only	530 (41)
Thrombolysis and EVT	484 (38)
Thrombolysis only	264 (21)
Recanalization (modified Thrombolysis in Cerebral Infarction >2b) (%)	
No	112 (9)
Yes	895 (70)
No angiogram available	271 (21)
Early neurologic improvement (%)	574 (45)
NA	26 (2)
Symptomatic intracranial hemorrhage (%)	34 (3)
NA	37 (3)
Intensive care unit admission (%)	128 (10)
NA	7 (1)
Modified Rankin Scale score at 90 d, %	
0	226 (18)
1	256 (20)
2	209 (16)
3	232 (18)
4	124 (10)
5	39 (3)
6	192 (15)
Days alive and out of hospital at 90 d (median [IQR])	71 [29, 84]

EVT indicates endovascular thrombectomy; and IQR, interquartile range.

**Figure 1:**
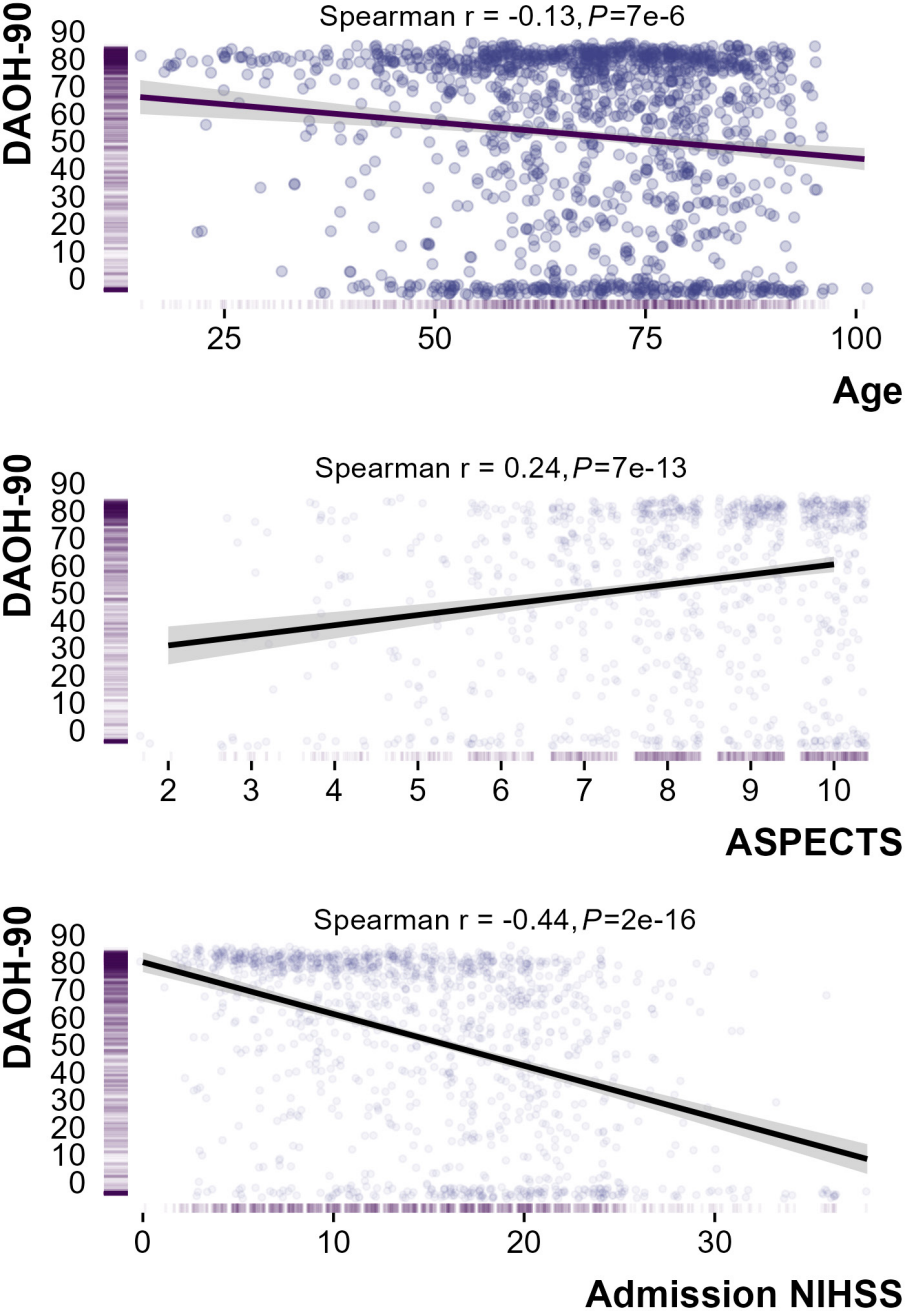
Correlations between DAOH‐90 and age of patients with hyperacute stroke (top), admission NIHSS score (middle), and admission ASPECTS. Increasing age, admission NIHSS score, and early ischemia (lower ASPECTS) were associated with lower DAOH‐90. ASPECTS indicates Alberta Stroke Program Early CT [Computed Tomography] Score; DAOH‐90, days alive and out of hospital at 90 d; and NIHSS, National Institute of Health Stroke Scale.

**Figure 2:**
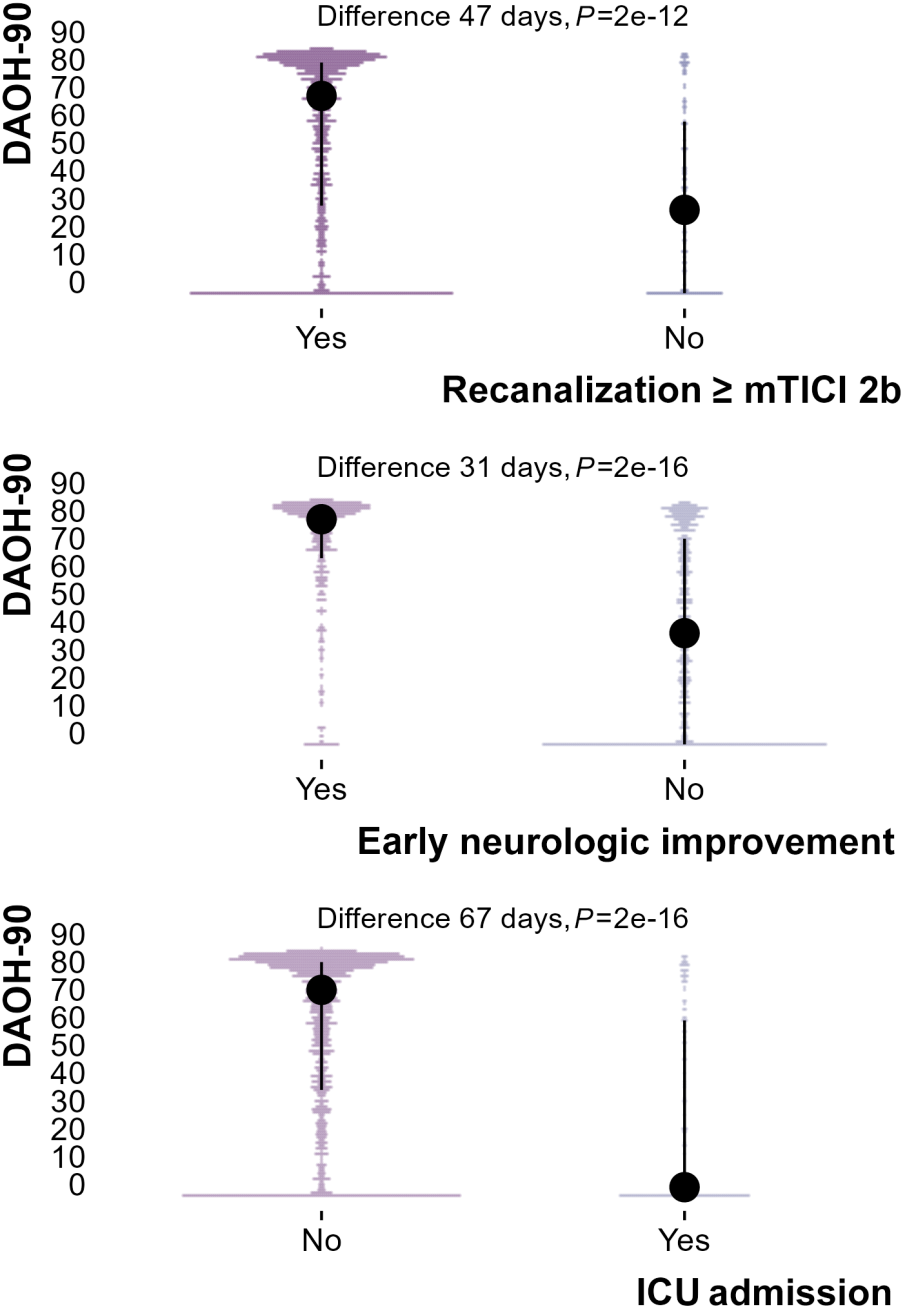
Impact of postadmission factors (recanalization status, early neurologic improvement, and ICU admission) on DAOH. Early treatment factors, change in clinical status, and requirement for high‐level support have significant impact on DAOH. DAOH‐90 indicates days alive and out of hospital at 90 d; ICU, intensive care unit; and mTICI, modified thrombolysis in cerebral infarction.

Increased DAOH was associated with younger age (Spearman rho −0.12, *P*=7e‐6), lower admission NIHSS score (Spearman rho −0.44, *P*=2e‐16) and higher ASPECTS (Spearman rho=0.24 *P*=7e‐13) (Figure 1). Intensive care admission reduced DAOH‐90 by 67 days (*P*<2e‐16, Figure 2) and early neurologic improvement increased DAOH by 31 days (*P*<2e‐16, Figure 2). In patients treated with EVT, successful recanalization was associated with a 47 day increase in DAOH‐90 compared with those without successful recanalization (*P*=2e‐12, Figure 2).

**Figure 3:**
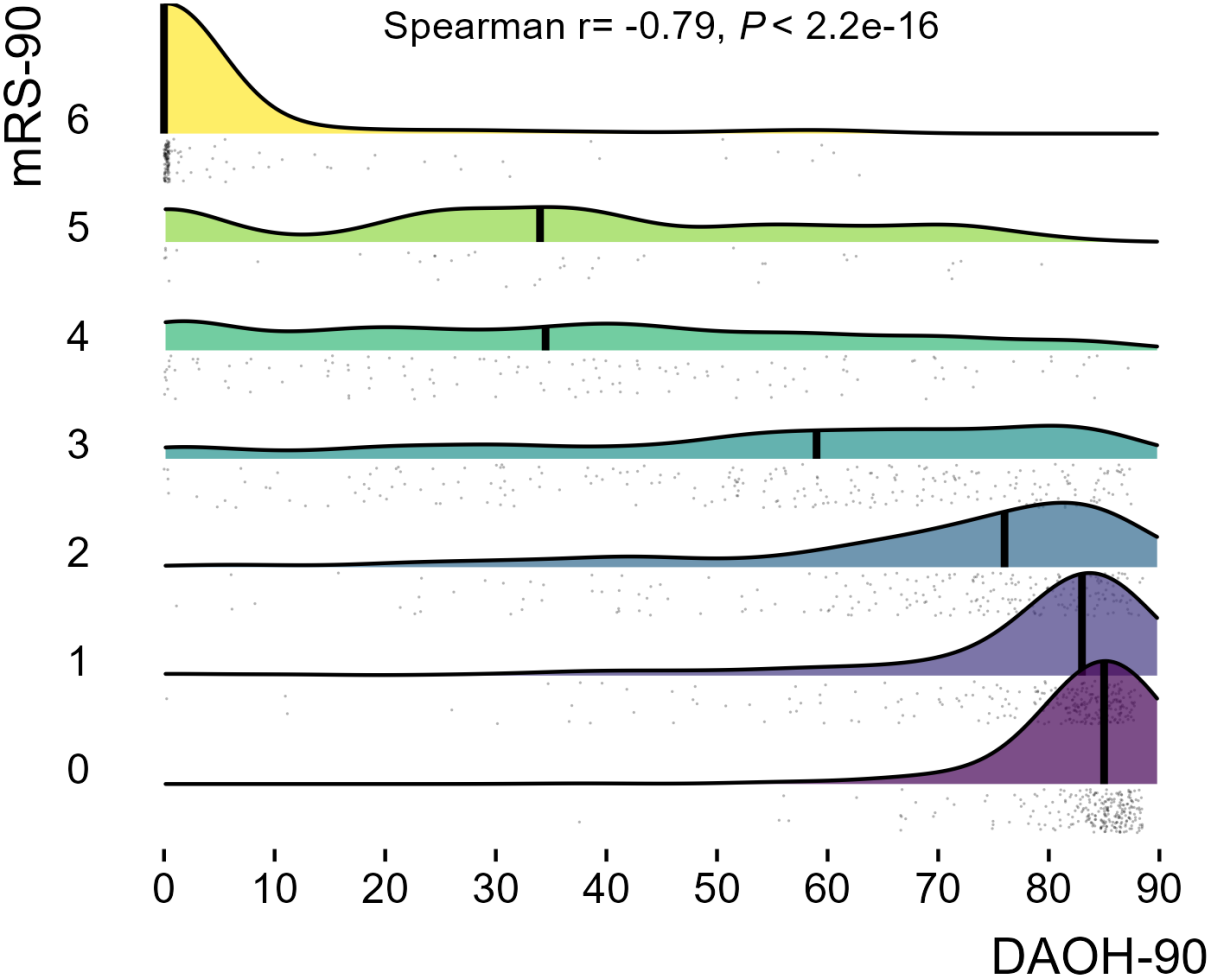
DAOH distribution stratified by mRS score. There is a significant correlation between increasing mRS‐90 and lower DAOH‐90. Note the wide distribution of DAOH especially in the mRS 3, 4, and 5 groups. DAOH‐90 indicates days alive and out of hospital at 90 days; and mRS‐90, modified Rankin scale at 90 days.

**Figure 4:**
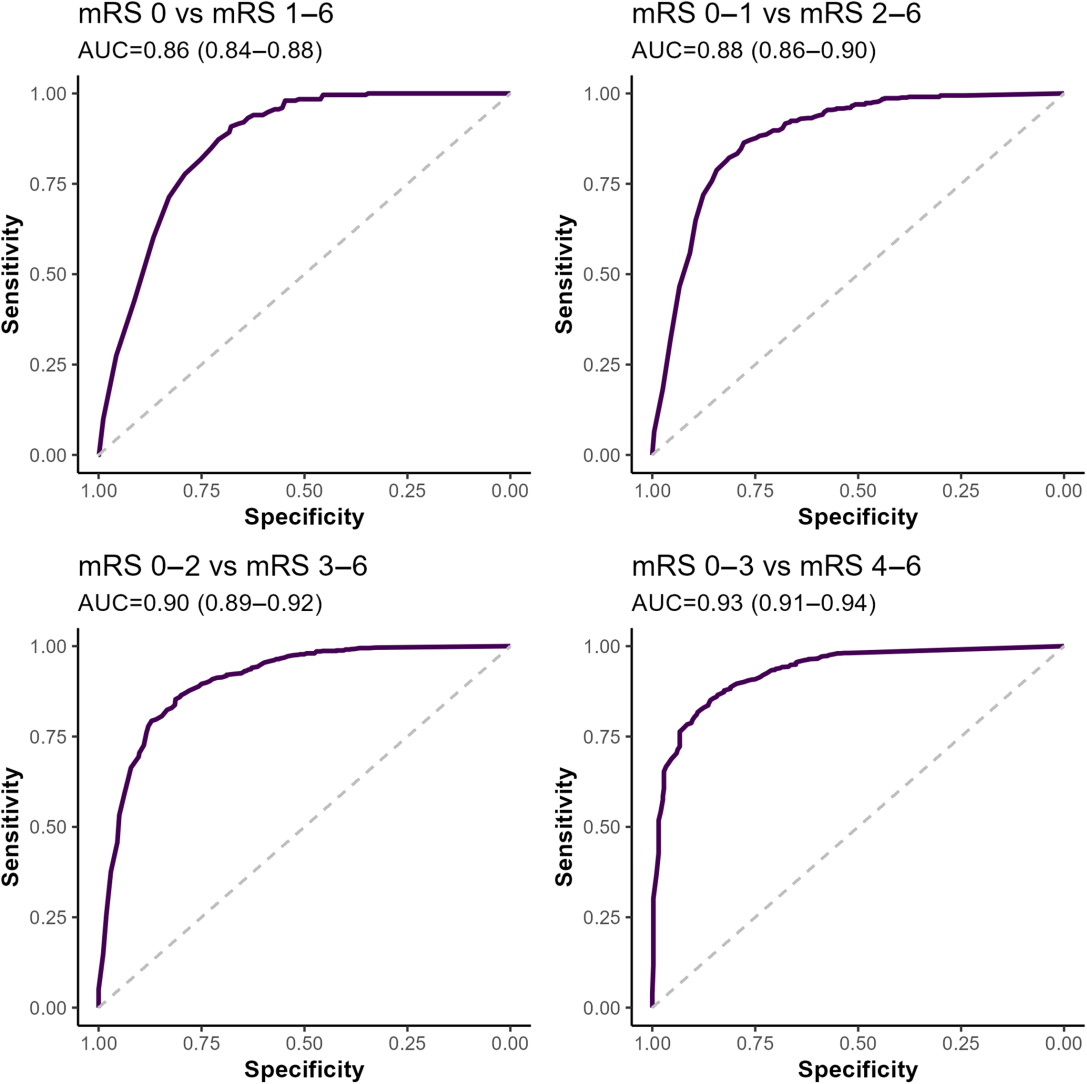
ROC analyses for distinguishing for ability of DAOH‐90 to distinguish between cutpoints of mRS‐90 (From top clockwise, mRS score 0 vs 1–6, mRS score 0–1 vs 2–6, mRS score 0–2 vs 3–6, mRS score 0–3 vs 4–6). AUROC progressively improved with a cutpoint at a higher level of mRS disability. AUROC, area under receiver operating curve; DAOH‐90, days alive and out of hospital at 90 days; mRS‐90, modified Rankin scale at 90 d; and ROC, receiver operating characteristic.

DAOH‐90 was strongly correlated with mRS‐90 (rho=−0.79, *P*=2e‐16, Figure 3). The distributions of DAOH‐90 within each mRS category were wide in mRS groups 3, 4, and 5. The receiver operating characteristic analysis showed a good ability of DAOH‐90 to discriminate between examined cutoffs of mRS: mRS score 0 versus 1–6, area under the curve 0.86 (95% CI, 0.84–0.88); mRS score 0–1 versus 2–6, area under the curve 0.88 (95% CI, 0.86–90); mRS score 0–2 versus 3–6, area under the curve 0.90 (95% CI, 0.89–0.92); and mRS score 0–3 versus 4–6, area under the curve 0.93 (95% CI, 0.91–0.94) (Figure 4).

The relative strength of association of patient outcome with demographic, clinical, and treatment prognostic factors was compared for DAOH‐90 and mRS‐90 (Table 2). Of the 14 prognostic factors tested, all 10 were significantly related to DAOH‐90 and 9 were significantly related to mRS‐90 score (Trial of Org 10 172 in Acute Stroke Treatment cause of large artery atherosclerosis was not statistically significant for mRS‐90 score).

**Table 2:** Relationship of Prognostic Variables With Median DAOH‐90 and Median mRS‐90

Factor	Median DAOH‐90	Difference	*P* value	Median mRS‐90	Difference	*P* value	Spearman correlation mRS vs DAOH	*P* value
Age, y								
≥70	66	−11	0.000158	3	1	1.40E‐10	−0.78	2.2E‐16
<70	77			2			−0.79	2.2E‐16
Sex								
Male	74	4	0.177	2	0	0.956	−0.81	2.2E‐16
Female	70			2			−0.77	2.2E‐16
Diabetes								
Present	54	−20	2.20E‐08	3	1	3.65E‐06	−0.81	2.2E‐16
Absent	74			1			−0.78	2.2E‐16
Ischemic heart disease								
Present	67.5	−4.5	0.255	3	1	0.0688	−0.81	2.2E‐16
Absent	72			2			−0.79	2.2E‐16
Atrial fibrillation								
Present	69	−5	0.129	2	0	0.084	−0.79	2.2E‐16
Absent	74			2			−0.79	2.2E‐16
Hypertension								
Present	67	−10	8.49E‐05	2	0	8.50E‐07	−0.81	2.2E‐16
Absent	77			2			−0.75	2.2E‐16
Cardioembolic TOAST cause								
Present	72	2	0.725	2	0	0.37	−0.78	2.2E‐16
Absent	70			2			−0.80	2.2E‐16
Large artery atherosclerosis TOAST cause								
Present	62	−11	0.0096	3	1	0.579	−0.82	2.2E‐16
Absent	73			2			−0.78	2.2E‐16
Admission National Institute of Health Stroke Scale score								
≥14	54	−28	2.20E‐16	3	2	2.20E‐16	−0.83	2.2E‐16
<14	82			1			−0.65	2.2E−16
Alberta Stroke Program Early CT [Computed Tomography] Score								
≥8	79	16	7.33E‐10	2	‐1	7.02E‐09	−0.74	2.2E‐16
<8	63			3			−0.82	2.2E‐16
Recanalization								
Present	71	47	1.52E‐12	2	−2	7.10E‐10	−0.78	2.2E‐16
(modified Thrombolysis in Cerebral Infarction ≥2b)								
Absent	24			4			−0.81	2.2E‐16
Early neurologic improvement								
Present	82	31	2.20E‐16	1	−2	2.20E‐10	−0.63	2.2E‐16
Absent	51			3			−0.81	2.2E‐16
Symptomatic intracranial hemorrhage								
Present	0	−74	1.30E‐13	6	4	2.52E‐14	−0.94	2.2E‐16
Absent	74			2			−0.77	2.2E‐16
Intensive care unit								
Present	7	−67	2.20E‐16	4	2	1.54E‐12	−0.89	2.2E‐16
Absent	74			2			−0.77	2.2E‐16

DAOH‐90 indicates days alive and out of hospital at 90 d; mRS, modified Rankin scale; and TOAST, trial of ORG 10172 in acute stroke treatment. For continuous variables, the median value was used for dichotomization.

The Spearman correlation between DAOH‐90 and mRS‐90 score was significant among all clinically relevant subgroups within the cohort (Table 2; Figure S4). In the patients with anterior circulation stroke receiving EVT, multivariable binary logistic regression models for mRS‐90 score ≤2 and DAOH‐90 score >70 demonstrated similar discriminative ability (c‐statistic 0.76 in both cases, Table S1).

In a post hoc exploratory analysis, the effect of recoding the DAOH‐90 score of patients who died some days after the stroke onset after leaving hospital to have a DAOH‐90 score of 0 resulted in an additional 39 patients (3% of total) shifting to a DAOH‐90 score of 0. The Spearman correlation between mRS‐90 and DAOH‐90 scores was similar with this adjusted DAOH‐90 score (Spearman rho −0.80 in the adjusted score versus −0.79 in the original score).

## Discussion

In patients with stroke treated with IVT and EVT, DAOH‐90 as a combined measure of hospitalization and mortality showed reasonable comparability to the established stroke outcome measure of mRS‐90. DAOH is an objective measure that can be determined from clinical and administrative data sets and its place in stroke trials warrants further study.

We would expect that DAOH‐90 score would decrease when previously described adverse prognostic factors are present. Therefore, the associations of DAOH score with age, admission NIHSS score, and ASPECTS provide validity evidence consistent with theoretical expectations.^20^ Likewise, a measure of outcome after stroke should ideally be sensitive to early indicators of treatment success and complications. In this context, patients without successful recanalization (11% EVT patients) or with intensive care unit admission (10% total patients) had lower DAOH values (Table 2; Figure 2).

The strong relationship of DAOH‐90 score with mRS score at 90 days in the full data set and subgroup analyses indicates that DAOH‐90 conveys similar information to the criterion standard, mRS. Using identical predictor variables, multivariable models for predicting dichotomized mRS‐90 (mRS score ≤2) showed similar discriminative ability in predicting dichotomized DAOH‐90 (DAOH score >70), again indicating reasonable comparability of DAOH‐90. Two smaller studies also reported a strong relationship of DAOH‐90 with mRS, with correlation coefficients of −0.73 for both studies.^10^, ^23^ In the current study, DAOH distributions were similar in patients with mRS scores of 0 and 1. Despite this, DAOH showed good performance in distinguishing the common mRS groupings of excellent outcome (mRS score 0–1 versus 2–6), functional independence (mRS score 0–2 versus 3–6), or more severe outcomes. This good discriminative ability of DAOH for the various dichotomies of mRS is likely contributed to by the clusters of low DAOH values in mRS 6 group and high DAOH values in the mRS 0 group. Although DAOH strictly measures mortality and hospitalization, the correlation with mRS may indicate that DAOH is influenced by factors such as disability, dependence, ambulation, and burden of care.

DAOH‐90 is reported as a continuous scale from 0 to 90 days and this allows finer gradation of outcomes compared with the more common ordinal or binary mRS measures such as excellent outcome, functional independence, or mortality. This has the potential for increasing statistical power to detect differences between interventions.^24^ However, the *P* values of association between established prognostic variables and DAOH‐90 were similar to that of mRS indicating DAOH 90 has similar statistical conclusion validity to mRS (Table 2). Although it is intuitive that an improvement in mRS score by even 1 point is clinically important, the minimal clinically important difference for DAOH‐90 is not immediately clear. This probably depends on the perspective: hospital resource allocation versus patient or clinician viewpoint.

DAOH offers several potential advantages over mRS as a stroke outcome measure. Although separate assessors can give different mRS scores for the same individual (in 1 study, agreement was only 43%^3^), DAOH does not require clinical judgments and therefore does not exhibit measurement error due to rater variability. DAOH assessment is also more convenient than assessment of mRS where a structured interview taking approximately 15 minutes is required for each patient,^3^ which incurs a significant cost for large studies. This convenience of DAOH is demonstrated by a large‐scale study that used a data set of 156 887 patients with acute ischemic stroke to determine hospital factors associated with increased home days (reduced DAOH).^25^ DAOH may allow for more complete outcome data sets by eliminating the need for a patient interview within a certain time frame. DAOH also allows for easy assessment of patient outcome at multiple time points, which may be particularly important given a quarter of patients with stroke have been shown to have late recovery between 3 months and 1 year.^26^ Finally, the fundamental unit (days) of DAOH is meaningful to patients and health professionals, for example at 90 days, patients treated with EVT spent 64 more days alive and out of hospital in the EXTEND‐IA (Extending the Time for Thrombolysis in Emergency Neurological Deficits–Intra‐Arterial) trial.^9^

DAOH as an outcome measure has a number of limitations. Time spent in intensive care, rehabilitation, and institutional care all represent different intensities of patient care but according to the simple measure of DAOH are treated with the same weight. In addition, a patient who remains in hospital for 85 days and lives past 90 days has the same DAOH score as someone who is discharged home after day 1 but dies 5 days later. To address this issue, some researchers code any death within follow‐up as 0^5^; however, this ignores any time spent at home before death, which may be considered of some value to a patient.^27^ Although DAOH‐90 provides a measure over 90 days, it has bimodal peaks at days 0 and 85 indicating more moderate DAOH values contribute less. For this reason, a recent study has used quantile regression with DAOH to assess differences in outcomes.^28^

There is high internal validity in our cohort, but external validation in other stroke populations needs to be performed. Only patients with stroke treated with IVT and EVT were included, and so the findings may be less applicable to patients not eligible for acute reperfusion therapies. The study was a retrospective analysis and so information as to why patients may have unexpected DAOH score could not be determined. We compared 2 outcome measures, neither of which are the “gold standard,” with mRS used as it is routinely collected and is one of the major outcome measures in stroke studies. There are other measurement systems like Patient Reported Outcomes Measurement Information System or Neuro‐QoL that are considered to be patient centered as they included patient input in development, measure constructs that many patients consider important, and responses represent the patient's perspective.^29^ Comparison of DAOH‐90 with other patient‐centered outcome measures should be performed.

## Conclusions

In patients treated with IVT or EVT, DAOH‐90 as a combined measure of hospitalization and mortality shows reasonable comparability to the more established stroke outcome measure of mRS‐90. DAOH‐90 can be readily obtained from administrative databases and therefore has the potential to be used in large‐scale clinical trials and comparative effectiveness studies. DAOH is an outcome measure that warrants further investigation.

## Sources of Funding

This study was supported by the Neurological Foundation of New Zealand and the Julius Brendel Trust.

## Disclosures

None.

## Supporting information

Data S1
