# Supplementary material for: Days Alive and Out of Hospital as an Outcome Measure in Patients Receiving Hyperacute Stroke Intervention
Source: J Am Heart Assoc. 2024 Jul 3;13(14):e032321. doi: 10.1161/JAHA.123.032321 (PMC11292750; doi:10.1161/JAHA.123.032321)
Supplement: Supplementary file 1 — Data S1 [file JAH3-13-e032321-s001.pdf]

SUPPLEMENTAL MATERIAL

Table S1. Multivariable model for predicting mRS and DAOH-90.

	mRS model	DAOH-90 model
**n**	871	871
**Dependent variable**	mRS ≤ 2	DAOH-90>70
**R-squared**	0.27	0.25
**C-statistic**	0.76	0.76

*Both models adjusted for age, admission NIHSS, baseline mRS, diabetes, thrombolysis, ASPECTS, and last known well to groin puncture time.

** Note, of the cohort of 1014 EVT patients, 133 patients were excluded as ASPECTS could not be calculated (posterior circulation stroke). A further 10 patients removed (5 with missing NIHSS, and 5 with missing pre stroke mRS).

Figure S1. Consort diagram for study.

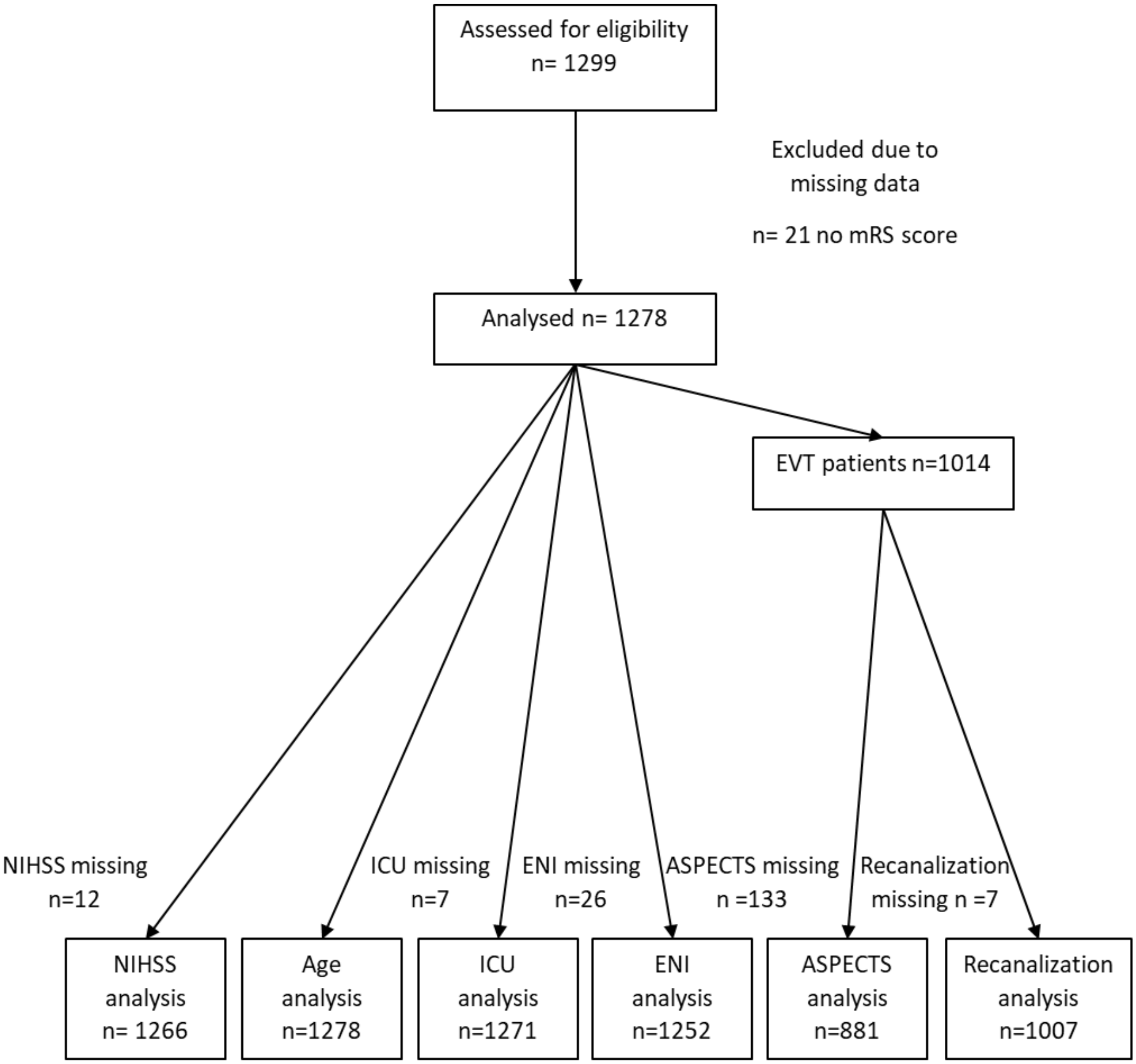

Note, for binary logistic regression models in supplementary material the ASPECTS cohort was used with a further 10 patients removed (5 with missing NIHSS, and 5 with missing pre stroke mRS).

mRS- modified Rankin scale; EVT – Endovascular thrombectomy; NIHSS – National Institute of Health Stroke Scale score; ICU – Intensive care unit; ENI – early neurologic improvement; ASPECTS – Alberta stroke programme early CT score

Figure S2. Distribution of DAOH-90.

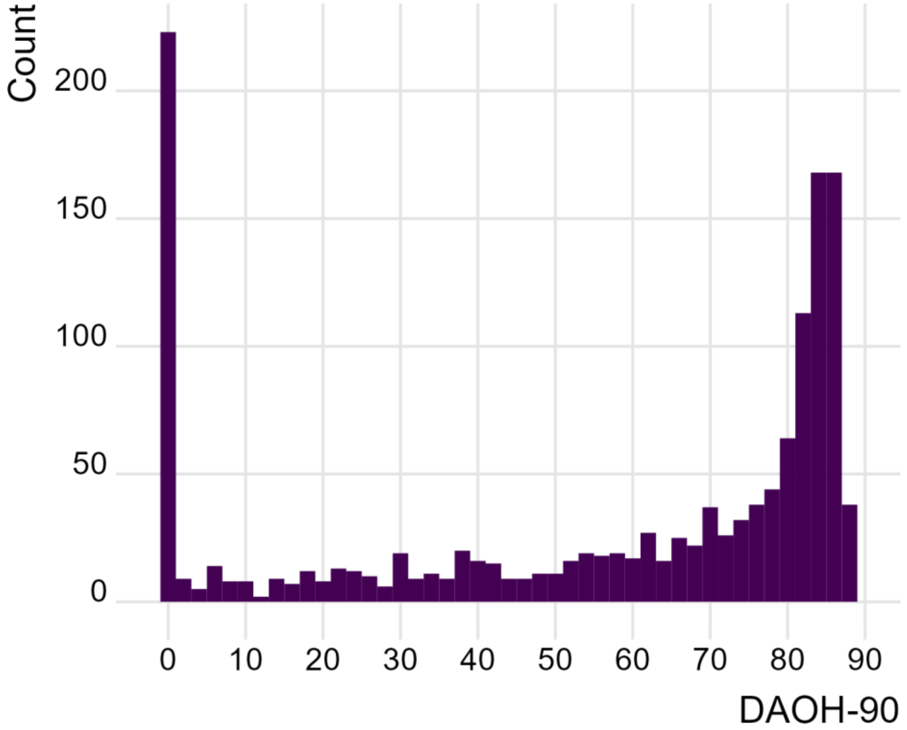

DAOH-90 shows a leftward skew bimodal distribution with a major peak at DAOH-90 of 0 days and a secondary DAOH-90 peak at 85 days. Median DAOH-90 was 71 days with a first quartile of 29 and a third quartile of 84.

DAOH-90 – Days alive and out of hospital at 90 days

Figure S3. Distributions of DAOH-90 across sub groups of age (top), admission NIHSS (middle) and admission ASPECTS (bottom).

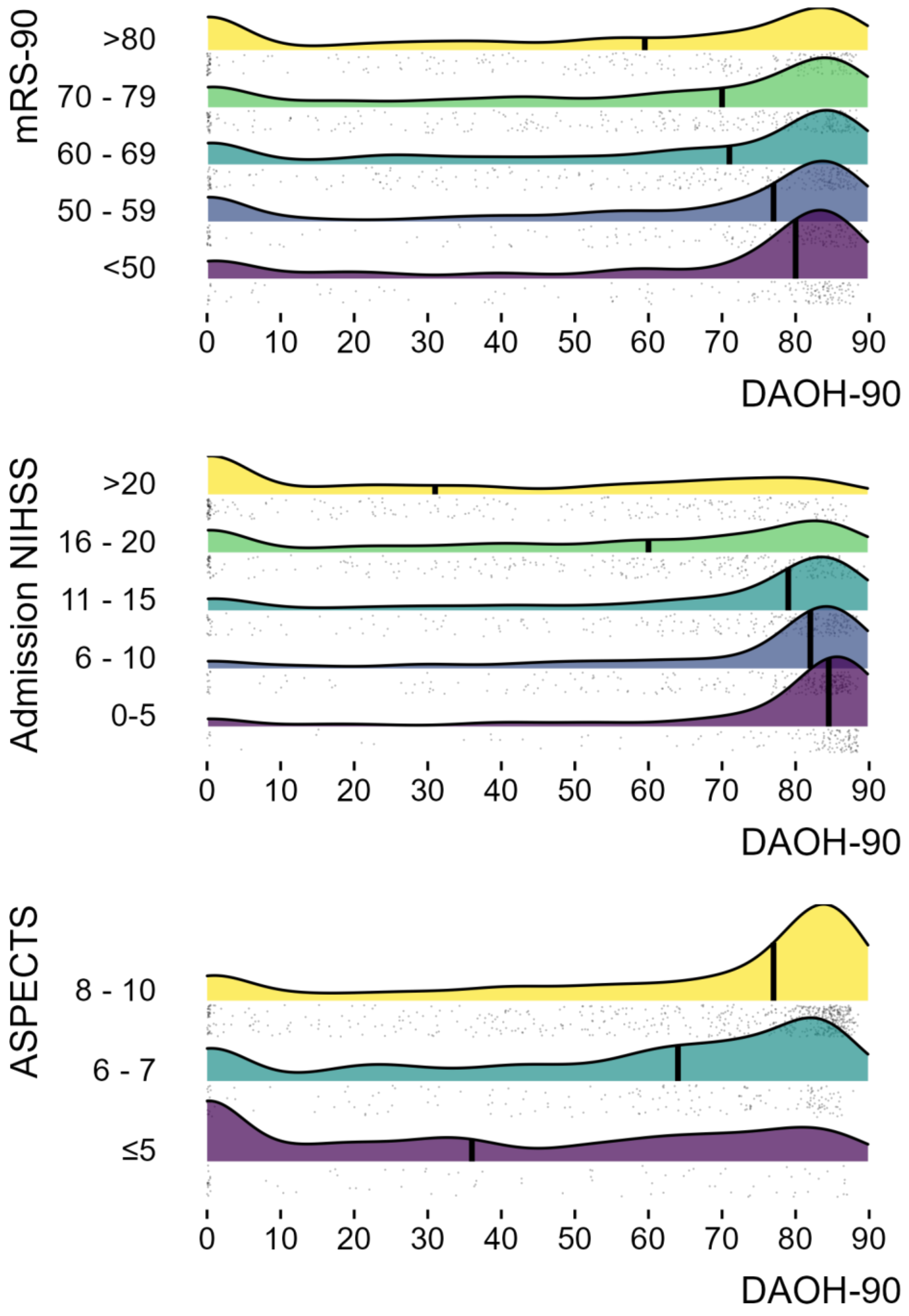

DAOH-90 – Days alive and out of hospital at 90 days; NIHSS – National Institute of Health Stroke Scale score; ASPECTS – Alberta stroke programme early CT score

Figure S4. Spearman correlation coefficients between DAOH-90 and mRS-90 across ethnic groups.

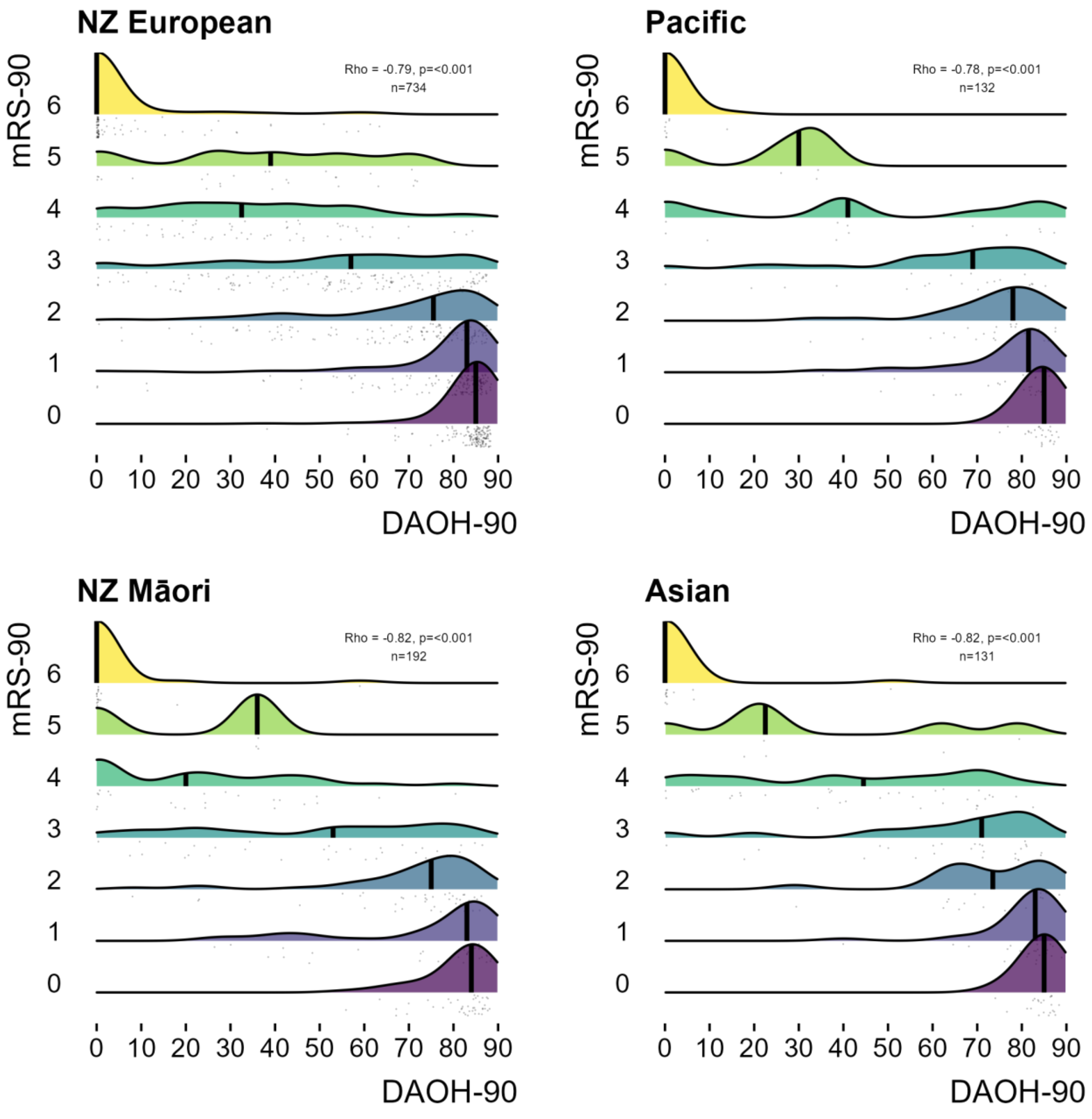

Similar strength correlations were observed across ethnic groupings.

DAOH-90 – Days alive and out of hospital at 90 days; mRS-90 – modified Rankin scale at 90 days
